# Management and Genetic Diversity of an Italian *Pistacia* Collection Through Microsatellite Markers

**DOI:** 10.3390/ijms27010013

**Published:** 2025-12-19

**Authors:** Nicola Santillo, Sabrina Micali, Ignazio Verde, Elisa Vendramin

**Affiliations:** Research Centre for Olive, Fruit and Citrus Crops (CREA-OFA), Council for Agricultural Research and Economics (CREA), Via di Fioranello, 52, 00134 Roma, Italysabrina.micali@crea.gov.it (S.M.); ignazio.verde@crea.gov.it (I.V.)

**Keywords:** *Pistacia*, EST-SSR, germplasm collection, genetic diversity

## Abstract

Conservation and characterization of germplasm collections are essential for safeguarding agrobiodiversity and supporting breeding programs. A collection of 140 accessions comprising three different *Pistacia* species, *P. integerrima*, *P. terebinthus*, and *P. vera*, was analyzed using 27 EST-SSR markers. On average, 3.4 alleles per locus, and 28.2% rare alleles were found. Observed heterozygosity (Ho = 0.36) was lower than expected (He = 0.48), while five loci displayed PIC values above 0.50, highlighting their high informativeness. The phylogenetic analysis clearly separated the three species. Among *P. vera* samples, Nj tree and population structure analysis identified three main sub-groups: Eastern Mediterranean/Middle Eastern accessions, Italian traditional cultivars, and US modern cultivars. The first group showed higher internal variability, reflecting both local diversification and historical genetic exchanges. Through the use of EST-SSR markers, the present study assesses the genetic diversity within the *Pistacia* collection while highlighting errors due to mislabeling issues. These results confirm the effectiveness of microsatellite markers to provide a framework for the management and exploitation of genetic diversity for breeding and conservation strategies, also in the *Pistacia* genus.

## 1. Introduction

The *Pistacia* genus belongs to the Anacardiaceae family and consists of more than 11 well-known tree and shrub species [1]; among them, *P. vera* is the only commercially significant species producing large edible nuts. *P. vera* is a dioecious, wind-pollinated, and diploid species with a haploid chromosome number n = 15 [2]. The center of origin of the pistachio is Central Asia, particularly northeastern Iran, Turkmenistan, and Afghanistan. Wild pistachio nuts dating back to the sixth millennium BC have been discovered in both Afghanistan and southeastern Iran, and natural pistachio forests are still found in these regions [3,4,5]. Pistachio subsequently spread from Persia to Mediterranean Europe through traders. In Italy, during the Roman Empire, it was known as the ‘Syrian nut’ [6,7]. At the end of the 19th century was introduced to the United States and Australia [6,7].

The United States and Iran, respectively, provide 51.85% and 23.62% of worldwide pistachio nut production (FAOSTAT, https://www.fao.org/faostat/en/#home accessed on 1 September 2025). The remaining 25% is grown in Syria, Turkey, and China. Italy has a very small production, but it is distinguished by a peculiarity of its nuts: the green color of the cotyledons. This variety, known as ‘Pistacchio di Bronte,’ is mainly used in pastry making [8,9]. The natural population of wild *P. vera* is centered in south-central Asia, which is expected to be the center of origin and diversification for the species [10]. Pistachio fruits were known as ‘nuts’ but are actually a semi-dry drupe composed of a yellow to green kernel enclosed in a hard shell (endocarp), and covered by a fleshy pericarp. Pistachio nuts are an important source of protein, fiber, monounsaturated fatty acids, minerals, and vitamins, as well as carotenoids, phenolic acids, flavonoids, and anthocyanins. Polyphenols in pistachios are essential to the antioxidant and anti-inflammatory properties, as demonstrated in vitro and in vivo through animal studies and clinical trials [11].

Pistachio trees have a long juvenile period spanning 5–10 years and a strong alternate bearing [12]. These two aspects greatly influence cultivation and production. Phenological and morphological characters are strongly affected by environmental conditions such as the chilling requirement, essential for fruit production [13,14,15]. Modern pistachio breeding programs have as main objectives to keep high productivity as constant as possible over time, together with improved qualitative traits and resistance to biotic and abiotic stresses. Despite breeding efforts in different parts of the world, most cultivars still bear undesirable characteristics such as a high percentage of unsplit, blank nuts, a very long unproductive or juvenile period, as well as extreme alternate bearing [10,16,17]. Among the biotic stresses that most affect *P. vera*, we can find soil-borne diseases and pistachio bushy top syndrome (PBTS). Soil-borne diseases are mainly caused by *Verticillium* and *Phytoptora* genera and more rarely by the *Armillaria*, *Rhizoctonia*, *Macrophomina*, and *Fusarium* [18].

Pistachio is a xerophytic species with good tolerance to saline and alkaline soils. For this reason, it might be used in the reforestation of arid and semi-arid areas of the planet. Nonetheless, climate change, causing the worsening of arid conditions in already arid areas, puts its great ability to the test. Finding genotypes that have resistance/tolerance to biotic and abiotic stresses is essential. The dioecism of the species is a crucial aspect that strongly affects breeding and selection of genotypes with desirable traits.

The chance to early select genotypes with desirable traits, without waiting for fruit production, is essential for fruit tree breeding programs to speed up the release of new selections and cultivars. The opportunity to follow the traits of interest without the need to evaluate phenotypes (Marker Assisted Breeding or Marker Assisted Selection) is crucial for fruit crops that are characterized by a long juvenile and unproductive period. Molecular markers associated with the interesting characters are a powerful tool to achieve this goal, and in the last twenty years, many studies have been performed on the *Pistacia* genus applying different molecular tools to obtain linkage maps, to study the genetic structure of *Pistacia* populations, and to characterize genetic materials and collections [19,20,21,22,23,24,25]. Among the different classes of molecular markers, Simple Sequence Repeats (SSRs) and Single Nucleotide Polymorphisms (SNPs) are considered the most useful. SSR markers are codominant, multiallelic, and scattered throughout the genome, and have been widely used in genetic diversity studies, population structure analyses, and identification of functional markers associated with important traits such as sex-associated markers in *P. vera* and wild *Pistacia* species [10,26,27,28]. In *Pistacia*, the use of a few SSR markers allowed sharp discrimination between male and female individuals, although their use remains limited to some species due to the lack of specific markers and a comprehensive understanding of the underlying genetic mechanisms [29,30]. On the other hand, SNPs are biallelic codominant markers and the most abundant in the genome. For these characteristics, they are widely used for genetic studies in many species, although in *Pistacia*, due to the late release of the genome sequence [31,32], SNPs are still not broadly applied [21,28,30,33]. Owing to their high genomic abundance and the reduced analysis costs enabled by a high level of automation, many studies report the advantageous use of SNPs compared to SSRs, especially in genome-wide association studies [33,34], as well as in sex discrimination [29,30]. By contrast, utilizing standard lab equipment, a narrow set of SSRs could be successfully used to study genetic diversity and to characterize germplasm collections and segregating populations [22,23,24,35,36].

In *Pistacia*, the application of Sequence Characterized Amplified Region (SCoT) markers and Kompetitive Allele Specific PCR (KASP) markers has been successfully reported. SCoT markers, which target specific regions of the genome and are highly reproducible, have been used for genetic diversity analysis and cultivar identification in *Pistacia* species [20]. Meanwhile, KASP markers, known for their high specificity and scalability, have emerged as promising tools for marker-assisted selection and sex identification in *Pistacia* [37]. Both marker types offer distinct advantages for improving genetic research and breeding programs in this genus.

Germplasm collections are an essential tool both to avoid the continuous erosion of agrobiodiversity and to allow the exchange and use of plant genetic resources [38,39], but they are susceptible to errors such as mislabeling and landraces renaming during their local diffusion, leading to synonymies (same genotype with different names) and homonymies (different genotypes with the same name) [38,40]. The level of duplication and errors within and among collections is known to be high, and the elimination of redundancy and errors is economically substantial [41,42]. Incorporating genetic characterization into Genbank management can solve these common issues and lead to more informed decisions and sustainable solutions. In 2006, the National Fruit Germplasm Collection (NFGC) of the Consiglio per la Ricerca in Agricoltura e l’analisi dell’Economia agraria—Centro Olivicoltura, Frutticoltura e Agrumicoltura (CREA-OFA) was established. The NGFC is the largest Italian fruit collection and the second in Europe, and, in the frame of the FAO International Treaty on Plant Genetic Resources for Food and Agriculture, the center maintains and characterizes around 5000 genotypes (two replicates each for a total of 9728 plants) belonging to more than 40 fruit species (pome fruits, stone fruits, nuts, small fruits, subtropical species).

In the present work, 27 EST-SSR markers out of 99, previously developed from the transcript of male and female inflorescences [43], were used to characterize, for the first time and extensively, the NFGC *Pistacia* germplasm collection to study the genetic diversity, the population structure, and to identify issues such as mislabeling and renaming. The present work highlights the effectiveness of microsatellite markers to routinely support Gen-Bank management in order to maintain and preserve genetic resources, avoiding resource waste and labeling, as well as introduction errors.

## 2. Results

### 2.1. EST-SSR Markers

Ninety-nine EST-SSR primer pairs, previously developed from male and female inflorescences of *P. vera* and only partially characterized [43], were initially tested on a small set of *Pistacia* samples composed of four males and four females, randomly chosen, belonging to *P. vera* and *P. terebinthus*, to assess amplification product presence and patterns.

A total of 50 markers out of 99 gave amplification products, and among them, 27 (Table 1) were polymorphic and easily scorable. The remaining 23 primer pairs were excluded because monomorphic or due to amplification patterns characterized by several stutter bands that prevented a robust identification of the alleles.

The 27 selected primer pairs were used for downstream genetic characterization of the *Pistacia* Collection of the NFGC. In total, 91 alleles were amplified, with the allele number ranging from two (EPVF018, EPVF030, EPVM002, EPVM017, EPVM033, EPVM035, EPVM040, EPVM043, EPVM050, EPVM054) to six (for EPVM049), with an average number of 3.37 alleles per locus. A proportion corresponding to 28.2% (31) of the total were rare alleles (frequency < 0.05%), and 26 private alleles were detected (Table S1). Private alleles were present mainly in *P. integerrima* (10), *P. terebinthus* (12), *P. integerrima × P. vera* ‘Chico’ (9), and only two private alleles were found in *P. vera* ’Rashiti’ and ‘Iraq’. The EPVF004 marker was the only monomorphic one, while EPVF023 was scored as multi-locus due to the presence of more than two alleles per locus with independent segregations.

The observed heterozygosity varied between 0.01 and 0.91 (EPVM033 and EPVM063, respectively) with an average of 0.36 per locus. This value was lower than the expected heterozygosity in all loci with the exception of EPVM002 and EPVM050. According to Botstein et al. [44], informativeness is limited for loci showing PIC values lower than 0.50. In this study, PIC values were equal to or above 0.50 for five loci (EPVM002, EPVM022, EPVM049, EPVM050, and EPVM054). The most informative markers were EPVM002 and EPVM050 with a PIC value of 0.53, while the least informative were EPVF013, EPVF018, EPVF030, and EPVM016 (PIC value of 0.43). The discrimination power (DP) showed an average of 0.33. The EPVM056 marker showed the highest discrimination power (PD = 0.89).

### 2.2. Genetic Diversity Analysis and Population Structure of the Pistacia Collection

**Figure 1:**
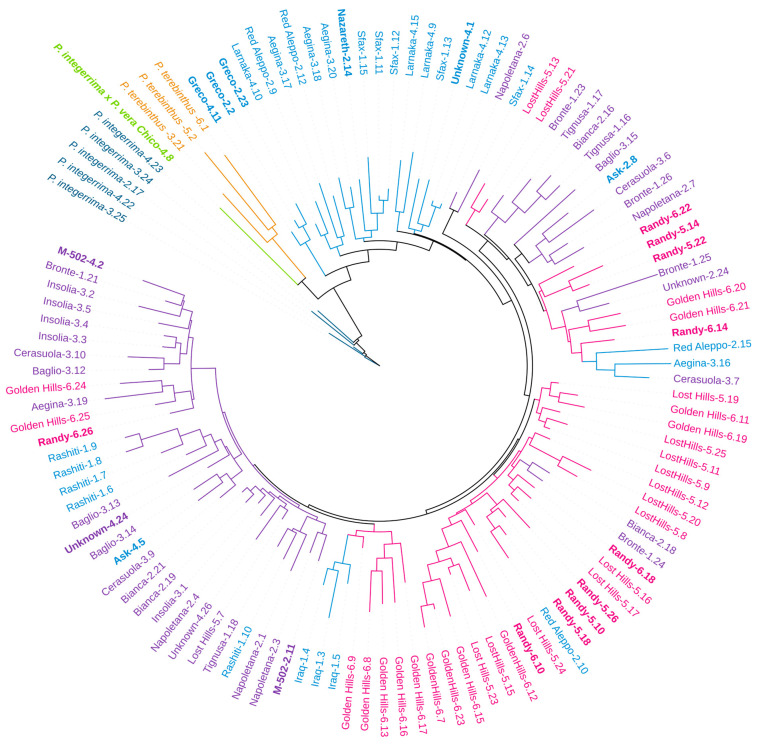
NJ phylogenetic tree obtained for 118 *Pistacia* genotypes. All male individuals are in bold, Italian genotypes are violet, Mediterranean accessions are light blue, USA cultivars are pink, *P. integerrima* are blue, *P. therebintus* are ochre, and *P. integerrima* × *P. vera* Chico are light green.

Samples were filtered to exclude accessions with more than 15% of missing data, and a total of 118 out of 140 accessions belonging to the NGFC *Pistacia* collection, comprising three different *Pistacia* species (Table S2), were retrieved and used for downstream applications. A neighbor-joining (NJ) phylogenetic tree was obtained based on a similarity matrix for the 118 samples, using 27 EST-SSRs (Figure 1).

The overall genetic similarity ranged from 0.47 to 0.98, with the lowest one recorded between *P. terebintus*-6.1 and *P. vera* ‘Ask’-2. Within *P. integerrima*, *P. terebinthus*, and *P. vera*, the genetic similarity ranges from 0.84 to 0.92, 0.73 to 0.77, and 0.60 to 0.99, respectively.

Mean genetic diversity parameters are reported in Table 2 for each of the three species. The observed heterozygosity (Ho) was highest in *P. integerrima*, and the lowest in *P. terebinthus*, and the polymorphic information content (PIC) was 0.51, 0.46, and 0.48 for *P. integerrima*, *P. terebinthus*, and *P. vera*, respectively.

The phylogenetic analysis clearly separated the three species, with *P. vera* and *P. terebinthus* being, as expected, the closest ones. *P. integerrima* and *P. terebinthus* had many species-specific alleles (Table S2), in particular seven characterizing *P. terebinthus* and six exclusive of *P. integerrima*. The diversity was also analyzed by a one-way perMANOVA analysis between the three species and the hybrid *P. integerrima* × *P. vera* sample. As expected, the pairwise comparisons between the three populations and the ‘Chico’ hybrid all showed *p*-values < 0.05 with Bonferroni correction (Table S3).

‘Chico’, a male chance pollinator introduced in the USA through Syria, clustered with the *P. terebinthus* genotypes. This accession shares 24% of its alleles with *P. therebintus* and *P. integerrima* and has two private alleles.

The remaining 109 genotypes belonging to *P. vera* were clustered together and grouped mainly based on their geographical origins. The NGFC *Pistacia* collection was composed of genotypes from the Mediterranean basin: Italy, Greece, Tunisia, Israel, Syria, and Cyprus. In addition, three USA cultivars, two female and one male, were present.

Most cultivars with a Middle Eastern origin, such as ‘Aegina’, ‘Red Aleppo’, ‘Sfax’, and ‘Larnaka’, were grouped together and separated from the Italian and USA genotypes. Interestingly, the samples named ‘Greco’ were grouped with the Mediterranean accessions, probably the name being an indication of the geographical provenience, and indeed, *Greco* is the Italian word for Greek (from Greece). The cultivars ‘Iraq’ and ‘Rashiti’ clustered together with the Italian accessions, and this unexpected result is a common situation observed in germplasm collections.

The traditional Italian cultivars were split into two sub-clusters reflecting their selection history and genetic connection [7]. All the Italian germplasm shares some peculiar traits, such as the accumulation of chlorophylls in the cotyledons, conferring a particular greenish color to the kernel, which makes them distinguishable throughout the world, and a longer and thinner shape compared to ‘Kerman’ [8,9].

The USA cultivars were grouped together with several Italian and Syrian accessions, and this could be explained considering the origin of Californian cultivars known to be developed from a restricted set of varieties imported into the USA at the beginning of the nineteenth century [45,46,47,48].

**Figure 2:**
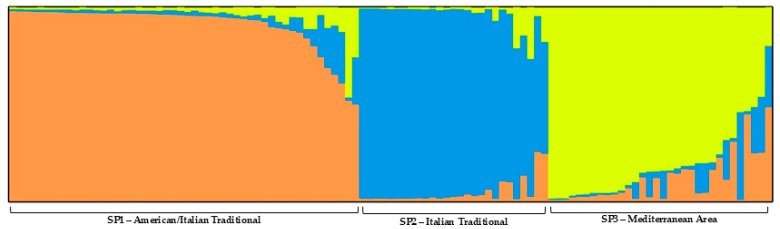
Population structure at k = 3. SP1—American/Italian group in orange, SP2—Italian traditional in blue, and SP3—Mediterranean area in yellow.

Population structure analysis was performed on 109 *P. vera* accessions. According to the Evanno method, the analysis revealed three subpopulations (K = 3; Figure 2). Considering the membership coefficient Q ≥ 0.80, 90 samples were clustered into three main subpopulations reflecting the NJ tree results: the SP1 was mainly composed of USA breeding genotypes and many Italian accessions, the SP2 comprised traditional Italian genotypes, and, in SP3, the largest parts of Mediterranean Basin accessions were grouped (Table S4). The remaining accessions (19) could not be assigned under the 80% membership coefficient criterion to any of the three subgroups and remained admixed.

The SP1 group (orange) is composed mainly of American cultivars (‘Golden Hills’, ‘Lost Hills’, ‘Randy’) with some Italian traditional genotypes (‘Bianca/Napoletana’, ‘Baglio’, ‘Bronte’, and ‘Insolia’). This is in accordance with Pistachio breeding history, since in the 1960s some Italian cultivars were introduced in northern California and used for several breeding programs enhancing overall quality [4,5]. The SP2 (blue) group of traditional Italian cultivars (‘Bianca/Napoletana’, ‘Insolia’, ‘Bronte’, ‘Tignusa’, ‘Baglio’, ‘Cerasuola’) all known to be indehiscent, with small fruits and the typical deep green kernels. Furthermore, ‘Bianca/Napoletana’ was known to be a population cultivar, and intra-genetic variability was expected. The SP3 group (yellow) was mainly composed of Mediterranean and Middle Eastern cultivars (‘Greco’, ‘Red Aleppo’, ‘Aegina’, ‘Sfax’, ‘Iraq’, ‘Larnaka’, and ‘Ask’).

## 3. Discussion

### 3.1. EST-SSR Markers

In the last two decades, several studies in *Pistacia* developed microsatellite makers, both from genomic DNA [24,29,35,49] and the expressed fraction of the genome [26,43], enhancing the knowledge of the genus and providing a genetic toolbox that is still relevant. SSR markers are still considered the markers of choice for this genus, due to the unavailability of SNP panels. Indeed, the first *P. vera* genome was recently published [31,32], but the sequences are not publicly available at the moment.

The high rate of unamplified primer pairs was also expected, given the intrinsic properties of the EST-SSRs. Microsatellite loci and the associated flanking regions, isolated from the expressed portion of the genome, could reside in the genomic DNA splicing sites or intron sequence entailing the unpairing of one or both primers. In this study, 54% of the primers did not give amplification products, and this is in accordance with other works. In particular, a comparative study on *P. vera* [24] tested 74 genomic SSR markers (SSRs) and 69 EST-SSRs. While 95% of SSRs were polymorphic and readily scorable and interpretable, only 57% of the EST SSRs displayed detectable levels of polymorphism. This confirms that EST-SSRs exhibit lower polymorphism than that observed in SSRs.

Four EPV markers are shared with a previous study [43], and allele number, Ho, and He are higher in the present work. This could be explained by the higher number of samples tested in this work (118) compared to the other study (only 20). On the contrary, the average number of alleles is lower compared to other studies that used genomic microsatellite markers [23,35], which reported a mean value per locus of 8.1 and 9.88, respectively. The PIC value is lower compared to other studies based on SSRs, but is higher in research using EST-SSRs, considering there are only three species in common [24,50]. These results could reflect the difference in the source of transcribed sequences and the techniques used for the isolation of the EST-SSR. In this study, markers were developed from *P.vera* male and female inflorescences by a subtractive hybridisation approach.

The reduced informativeness of EST-SSR markers compared to genomic SSRs can be explained by considering the higher conservation rate of transcribed regions compared to the genomic ones. EST-SSRs tend to be more conserved and functionally constrained, reducing the level of polymorphism, because they come from coding sequences. In contrast, genomic SSRs, especially those located in non-coding regions, are less subject to selective pressure, allowing for greater allelic variability.

### 3.2. Genetic Diversity Analysis and Population Structure of the Pistacia Collection

#### 3.2.1. Genetic Diversity Analysis

The genetic analysis conducted using EST-SSRs highlighted a clear separation of each accession and a clustering based on the species (*P. integerrima*, *P. therebintus*, and *P. vera*) and the geographical origins for *P. vera*. These results are in agreement with other studies [35,50] reporting a well-defined separation of the different *Pistacia* species using both EST-SSR and SSR molecular markers. A study on 24 *P. vera* samples using 206 SSR markers reports genetic diversity parameters close to the results of the present study, particularly 0.46, 0.55, and 0.50 for Ho, He, and PIC, respectively [35]. In comparison to other research conducted using EST-SSRs reporting Ho = 0.38, He = 0.40, and PIC = 0.34 [50], the mean values of the present work show a higher degree of genetic diversity in the *P. vera* samples. As reported in previous works [19,51,52], *P. integerrima* was the last to diverge among the three species analyzed, while *P. vera* was considered the more primitive species of the genus [1,53]. These results were supported by the observation of the highest *P. vera* diversity in the center of diversity in Central Asia [20,54,55]. *P. integerrima* and *P. terebinthus* were both rootstocks used in *P. vera* orchards, and *P. terebinthus* had been used as a pollinator in the past, again supporting the phylogenetic classification. The high genetic similarity observed inside the *P. terebinthus* and *P. integerrima* groups is expected since these samples came from a nursery and are likely to be seeds derived from the same progenitor.

The cluster localization of the male accession ‘Chico’ supports the assumption, based on its morpho-phenology (leaf characters and bloom period), that it was probably an interspecific hybrid between *P. vera* and *P. integerrima* [5,45,56].

The observed separation of *P. vera* genotypes is in agreement with different studies that clearly separate *Pistacia* genotypes based on their geographical origins [24,35,50]. The Mediterranean group, composed of accessions coming from Greece, Tunisia, Israel, Syria, and Cyprus, shows a higher level of genetic diversity in comparison with the Italian ones. This result could reflect their lower distance from the center of diversity of the species located between Turkmenistan and Iran [4].

The USA cultivars were grouped with the Italian and Syrian accessions, and this can be explained considering the origin of Californian Pistachio breeding programs started at the beginning of 1900 in the USDA Plant introduction garden at Chico (CA, USA), by introducing a narrow number of cultivars, less than 20, from Italy, Syria, IRAN, Tunisia, Greece and Israel [45]. In particular, the traditional Italian cultivars, ‘Bronte’ and ‘Trabonella’, and ‘Red Aleppo’ from Syria were introduced in that collection and used as pollen donors [4]. Starting from the 1960s, Californian breeding programs released some of the major cultivated pistachio cultivars such as ‘Kerman’, ‘Golden Hill’, ‘Lost Hill’ [5,45,46,47,48]. The low genetic diversity of these cultivars reflects the founder effect, due to the low number of initial accessions utilized in breeding programs [56].

Regarding the accessions with the same name that did not belong to the same clade, the history of the NFGC, planted 30 years ago, can explain these results, as some genotypes were introduced as seeds instead of grafting scions for clonal propagation. Germplasm collections are prone to errors such as mislabeling and landraces renaming during their local diffusion, resulting in synonymies (same genotype with different names) and homonymies (different genotypes with the same name). The level of duplication and errors within and among collections is known to be high [38,40,41,42]. This is the case for the cultivars ‘Iraq’ and ‘Rashiti’ that clustered together with Italian accessions. In particular, in the case of ‘Rashiti’, a mislabeling of the bud sticks could be the likely explanation. Indeed, the *Pistacia* germplasm collection of the NFGC of CREA-OFA was transferred from its original site to the current location in the early 2000s, and errors may have occurred during the relocation process. Moreover, the so-called ‘Iraq’ genotypes clustering with the Italian accessions, despite their name, were introduced to the NGFC from Sicily (historical register and Dr. Avanzato’s personal communication), collecting scions from a tree that was known to be from IRAQ. This is a typical event that introduces inconsistency in germplasm collections; moreover, no commercial cultivar bearing this name is known. Such variability may reflect the maintenance of different local selections under the same varietal name or the occurrence of spontaneous hybridizations within germplasm collections [54] as well as the presence of cultivar populations [57] like ‘Bianca/Napoletana’ cultivar. In this case, internal genetic variability was expected.

#### 3.2.2. Population Structure

Population structure partially overlaps with the cluster analysis outcomes. The presence of three, well-defined genetic groups based on the geographic origin of the accessions is in accordance with other studies conducted on *P. vera* and related species [6,19,58].

The population structure and the phylogenetic analysis also reflect the differences in pistachio drupes characterizing different areas. Italian cultivars typically have a very deep green kernel and a distinctive, high-quality flavor. However, their high percentage of indehiscent endocarps makes them less suitable for fresh consumption, while they are highly valued in the food industry [8,59]. Middle Eastern cultivars generally show a high percentage of split nuts (though not as high as American cultivars) and a kernel color that becomes lighter green as maturity progresses, except for ‘Sfax’, which is characterized by a greater variability in color intensity. American cultivars combine the most desirable traits in terms of dehiscence and nut size [56]. Their kernels are larger than those of other groups, but their flavor and color are typically less intense.

#### 3.2.3. Germplasm Management

Germplasm collection management can greatly benefit from genetic characterization in terms of reducing unwanted redundancy, mislabeling, and spelling errors. SNP and SSR markers are the most commonly used for these purposes. SNPs are the most abundant in the genome and can be automated, but due to their biallelic nature, a high number of loci must be screened. On the contrary, SSR markers are multi-allelic with more than 10 alleles at a single locus (up to 19 in this study) and require a much smaller number of loci to be tested for fingerprinting and diversity studies. SSR markers are easily manageable by small laboratories.

In the present study, a set of 27 EST-SSRs developed from the transcriptome of male and female *P. vera* inflorescences proved to be effective in discriminating inter- and intra-Pistacia genus samples. The efficiency of this marker set allowed for the clear separation of the *P. integerrima* × *P. vera* ‘Chico’ interspecific hybrid.

## 4. Materials and Methods

### 4.1. Plant Materials and DNA Extraction

Analyses were carried out on 140 *Pistacia* accessions (Table S1) from the National Fruit Germplasm Centre of CREA-OFA in Rome: 116 *P. vera*, 15 *P. integerrima*, 6 *P. terebinthus*, 2 unknown genotypes, and one *P. vera* × *P. integerrima* hybrid (40A). The *Pistacia* collection is composed of 20-year-old trees grown at the NFGC of CREA-OFA in Rome (41°47′42″ N, 12°33′46″ E). Samples were named with the name of the species/variety/accession followed by the position in the field (row and number of the tree in the orchard), to keep track of the different accessions carrying the same name.

Young leaves and apices were collected, and after being frozen in liquid nitrogen, they were stored at −80 °C. DNA was then extracted by grinding the vegetal material with liquid nitrogen, using the Genomic DNA Mini Kit (Plant) Protocol (Geneaid; 221, New Taipei City, Taiwan ) with minor modifications. DNA quantification and integrity were evaluated both by gel electrophoresis (agarose concentration of 0.8%) and NanoDrop 1000 Spectrophotometer (Thermoscientific; Waltham, MA, USA). DNA samples were diluted to a final concentration of 10 ng/µL.

### 4.2. SSR Analysis

A total of ninety-nine EST-SSR markers, previously developed from male (EPVM) and female (EPVF) inflorescences, were evaluated in eight randomly selected *Pistacia* samples to assess the presence of amplification products. Based on their amplification profile, twenty-seven markers were chosen and tested on the 140 accessions of the NFGC.

Amplifications were performed in a final volume of 10 μL using, for each reaction, 10 ng of genomic DNA, 1.5 mM MgCl2, 0.2 mM dNTPs, 0.1 μM of each primer, 5 U/μL of Platinum^®^ Taq DNA Polymerase (Invitrogen; Waltham, MA, USA), and the following amplification program:
Denaturation at 95 °C for 5 min;30 cycles at 95 °C for 30 s;Annealing temperature for 30 s,Extension 72 °C for 30 s;Final extension at 72 °C for 30 min.
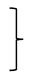
30 cycles;

Primers were labeled with three different dyes, and the amplification reactions were diluted according to the intensity of the fluorescence (1:10 D4-PA dye (blue), 1:5 for D3-PA dye (green), and no dilution for the D2-PA dye (black), (AB SCIEX, Framingham, MA, USA).

The amplification products were separated using a capillary electrophoresis apparatus (CEQ 8800 EX, AB SCIEX; Framingham, MA, USA). For each sample, 0.5 μL of 608098 DNA Size Standard Kit 400 bp (AB SCIEX; Framingham, MA, USA) was loaded as an internal standard. PCR products with fragment size > 350 bp were separated by horizontal electrophoresis using a high-resolution agarose gel MetaPhor (Cambrex; East Rutherford, NJ, USA) with a concentration of 2% or 3% based on the expected fragment size. A constant voltage of 5 V/cm was applied, and the fragments were visualized by staining with Ethidium Bromide on a Gel Doc XR + (Biorad; Hercules, CA, USA) apparatus.

The allele size estimation was performed by comparing the amplification products with the TriDye™ Ultra Low Range DNA Ladder (New England Biolabs; Ipswich, MA, USA) for the agarose gel and by the Genetic Analysis System (CEQ System A16638-AA April 2004, AB SCIEX; Framingham, MA, USA) for the capillary electrophoresis. To improve scoring accuracy and robustness, two independent readings for each EST-SSR marker were taken.

### 4.3. Genetic Diversity Analysis

The allele number per locus (Na), the effective number of alleles per locus (Ne), the number of rare (frequency < 0.05) and unique or private alleles (specific to a genotype), the observed and expected heterozygosity (Ho and He), the polymorphic information content (PIC), the discrimination power (DP) of each marker were calculated using iMEC: Online Marker Efficiency Calculator [60]. The PAST 5.2.1 [61] software package was employed to estimate genetic relationships among cultivars. In particular, a neigbour-joining phylogenetic tree [62] was built using a similarity matrix obtained with DICE as the similarity index, and a post hoc pairwise PerMANOVA (Multivariate ANalysis Of VAriance) test between the three groups and the hybrid sample (*P. integerrima* × *P. vera*) was performed. Pairwise comparisons (at *p* < 0.05) are Bonferroni corrected.

### 4.4. Population Structure Analysis

Population structure analysis was carried out by the Structure 2.3.4 software [63] based on Bayesian statistics using 27 SSR markers. The admixture model of ancestry and correlated allele frequencies was adopted to analyze the dataset, with no preliminary subpopulation information.

The proportion of the ancestry of each individual was tested, considering a K number from 1 to 10, with 10 iterations for each value of K. The settings for burning-in and MCMC (Markov Chain Monte Carlo) were 10,000 and 100,000, respectively. To determine the K number, the model established by Evanno et al., 2005 [64], was adopted using the software CLUMPAK (http://clumpak.tau.ac.il/ (accessed on 16 December 2025); Clustering Markov Packager Across K) [65].

## 5. Conclusions

This work represents a comprehensive EST-SSR characterization of the *Pistacia* collection conserved at the NGFC, maintained in the experimental fields of CREA-OFA in Rome. The set of 27 markers was used for the first time on 118 *Pistacia* samples. The molecular characterization enabled the estimation of the genetic diversity of the collection and the building of a *Pistacia* database useful for fingerprinting purposes. Despite the lower level of information of transcriptome-derived markers compared to genomic ones, EST-SSRs were effective in distinguishing accessions, detecting private alleles, and resolving varietal relationships within and across species. Of particular interest was the identification of species-specific private alleles and the discrimination of the hybrids.

Within the *P. vera* samples, three main clusters were identified that reflected the geographical origin and the breeding history, and some peculiar kernel traits of the materials were assayed. The clustering of the accessions into three main genetic groups confirms the separation of American, Eastern Mediterranean/Middle Eastern, and Italian germplasm, while also revealing patterns of admixture resulting from historical exchanges and conservation practices. The distinct genetic heterogeneity observed among Italian cultivars underscores their potential as a valuable reservoir of diversity for future breeding programs. Overall, this study contributes to the safeguarding and valorization of *Pistacia* genetic resources and provides a molecular framework that can support breeding strategies aimed at improving resilience to biotic and abiotic stresses.

Despite the discrimination power of EST-SSRs being lower than genomic-derived SSRs, the molecular marker set tested could efficiently empower the management of germplasm collections, reducing unwanted redundancy, mislabeling, and spelling errors. The availability of the *Pistacia* genome sequences could lead to the isolation of new SSRs that can be in silico tested and the development of an SNP panel to advance the genetic knowledge on the genus.

## Figures and Tables

**Table 1:** Genetic diversity of 118 *Pistacia* accessions analyzed by 27 SSR markers. Locus name, amplification range, number of alleles per locus (Na), observed heterozygosity (Ho), expected heterozygosity (He), polymorphic information (PIC), Power of Discrimination (D), and number of alleles with MAF < 0.05.

Marker	Range	Na	Ho	He	PIC	D	MAF < 0.05
EPVF010	208–216	3	0.03	0.46	0.46	0.03	2
EPVF013	570–670	3	0.64	0.5	0.43	0.7	1
EPVF018	700–720	2	0.06	0.5	0.43	0.75	--
EPVF021	219–228	3	0.1	0.56	0.48	0.23	1
EPVF023a	191–205	4	0.17	0.41	0.48	0.37	--
EPVF023b	217–245	4	0.11	0.42	0.48	0.08	2
EPVF030	600–610	2	0.04	0.52	0.43	0.04	1
EPVF032	610–680	3	0.88	0.47	0.44	0.61	1
EPVM002	635–645	2	0.79	0.21	0.53	0.19	--
EPVM016	490–500	3	0.56	0.5	0.43	0.72	--
EPVM017	245–249	2	0.04	0.64	0.47	0.33	--
EPVM022	238–250	5	0.09	0.37	0.5	0.09	2
EPVM024	227–248	5	0.03	0.53	0.49	0.34	4
EPVM032	220–245	5	0.75	0.52	0.47	0.31	2
EPVM033	302–304	2	0.01	0.67	0.48	0.45	1
EPVM035	140–143	2	0.19	0.53	0.46	0.26	--
EPVM040	107–116	2	0.07	0.61	0.47	0.3	--
EPVM041	307–330	4	0.07	0.44	0.49	0.11	3
EPVM043	940–950	2	0.39	0.49	0.49	0.34	--
EPVM049	249–291	6	0.29	0.38	0.5	0.13	3
EPVM050	920–940	2	0.72	0.24	0.53	0.21	--
EPVM051	110–149	4	0.76	0.56	0.46	0.34	1
EPVM054	900–950	2	0.58	0.38	0.51	0.32	--
EPVM056	405–425	5	0.59	0.44	0.45	0.89	1
EPVM058	245–275	4	0.05	0.49	0.49	0.19	2
EPVM059	220–245	5	0.66	0.57	0.48	0.39	2
EPVM063	157–190	5	0.91	0.67	0.49	0.19	2
All	107–950	91	0.36	0.48	0.47	0.33	1.15

**Table 2:** Overall genetic diversity for the three *Pistacia* species analyzed.

	Na	Ho	He	PIC	D
*P. terebinthus*	1.75	0.42	0.41	0.46	0.65
*P. integerrima*	1.82	0.61	0.41	0.51	0.5
*P. vera*	2.61	0.44	0.47	0.48	0.31

## Data Availability

The original contributions presented in this study are included in the article/Supplementary Materials. Further inquiries can be directed to the corresponding author.

## Supplementary Materials

The following supporting information can be downloaded at: https://www.mdpi.com/article/10.3390/ijms27010013/s1.

## Abbreviations

The following abbreviations are used in this manuscript:

NGFC CREA	National Fruit Germplasm Collection, Council for Agricultural Research and Economics
ITPGRFA	International Treaty for Plant Genetic Resources for Food and Agriculture
GWAS	Genome Wide Association Studies
EST-SSR	Expressed Sequence Tag Simple Sequence Repeat
EPVM/F	Expressed sequence tag Pistacia Vera Male/Female
SSRs	Simple Sequence Repeats
SNPs	Single Nucleotide Polymorphisms
PD	Discrimination Power
Ho	observed Heterozygosity
He	expected Heterozygosity
PIC	Polymorphic Information content
PI	Probability of Identity
MAF	Minor Allele Frequency
